# Exploring dietary patterns during school hours in Swedish adolescent student population, focusing on out-of-school eating and snacking: a repeated cross-sectional study

**DOI:** 10.1186/s12889-026-29555-8

**Published:** 2026-09-22

**Authors:** Hanna Wieslander, Sofia Spolander, Vasileios Papapanagiotou, Alexandros Papadopoulos, Emma Patterson, Ioannis Ioakimidis

**Affiliations:** 1 Department of Medicine, Karolinska Institutet, Huddinge, Stockholm, Sweden; 2 Arbisense AB, Stockholm, Sweden; 3 Department of Global Public Health, Karolinska Institutet, Stockholm, Sweden; 4 Division for Risk and Benefit Assessment, Swedish Food Agency, Uppsala, Sweden

**Keywords:** Adolescents, Dietary behaviours, School lunches, Food environment, Fast-food access, Snacking, School lunch participation

## Abstract

**Background:**

Establishing healthy dietary habits during childhood and adolescence is essential, as these patterns often persist into adulthood and influence long-term health. In Sweden, school lunches play an important role in overall nutritional intake. However, among adolescents, school lunch participation may decrease, while purchasing food from nearby retailers becomes increasingly common. This study aims to investigate dietary patterns among adolescents aged 13–19 years in Stockholm, Sweden, during school hours, focusing on school lunch participation and snacking inside and around school.

**Methods:**

A repeated cross-sectional study was conducted over four years, with independent student samples recruited each year from two schools in central Stockholm, an area with high density of fast-food retailers. Data were collected in 2019–2021 and 2024 and included self-reported pictures of meals and beverages, and self-rated dietary habits. Images were collected through school-based citizen science projects using purpose-built mobile apps that recorded GPS-tagged pictures, indicating eating locations. Manual image annotations were used for nutritional assessment.

**Results:**

Four hundred eighty-three participants were included, contributing 4,562 images. In total, 21% of meals and drinks were reported outside school grounds. The proportion of unhealthy meals was significantly higher outside than inside school (65% vs. 43%, χ^2^ = 109.6, *p* < 0.001). Meals consumed inside school more often comprised components of a complete meal and contained significantly higher proportions of vegetables, red and processed meat, and fish and seafood (all *p* < 0.001) compared with meals eaten outside school, which were more often snack-based. Sweetened beverages were more frequently reported outside school (*p* < 0.001 for both sugar- and artificially sweetened beverages).

**Conclusion:**

This study shows that despite access to free school meals, students in central Stockholm tend to leave school to consume less healthy lunches outside school. Combined with the easy access to vendors selling unhealthy foods and the frequent consumption of unhealthy snacks both in and outside school, these patterns highlight important public health concerns related to the school food environment. Overall, this study provides new insights into adolescents’ dietary patters during the school day, providing a foundation for future research and interventions supporting healthier eating among students.

**Supplementary Information:**

The online version contains supplementary material available at https://doi.org/10.1186/s12889-026-29555-8.

## Background

Poor dietary habits are a major contributor to the global burden of disease and an important risk factor for several non-communicable diseases [1]. In Sweden, childhood obesity is a growing public health concern, with more than one in five individuals aged 16–19 today living with overweight or obesity [2].

Despite the central role of diet in preventing these diseases, most children in Sweden do not meet the dietary recommendations [3]. According to the Nordic Nutrition Recommendations, a healthy and sustainable diet includes a high intake of foods such as vegetables, fruits and whole grains, while limiting the intake of red and processed meat and foods rich in fat, salt and sugar [4]. However, in Sweden, most children and adolescents do not eat enough fruit and vegetables, exceed the recommendations for red and processed meat consumption, and have a high consumption of sweets, savoury snacks and sugary drinks [3].

Establishing healthy dietary habits early in life is critical, as these patterns often persist into adulthood and influence the overall health [5]. Schools play an important role in establishing healthy dietary habits, reaching a large part of the population [5]. Previous studies have shown the importance of school lunches as a contributor to the overall intake of healthy foods among school children [6–9]. On weekdays, school lunches account for approximately a quarter of children’s overall energy intake and are generally more nutrient-dense and less energy-dense than meals eaten outside of school grounds [9].

However, during adolescence (10–19 years of age), students become increasingly independent in their food choices and are more strongly influenced by the surrounding food environment [10]. During this period, school lunch skipping and purchasing food outside school may also become more common [11, 12]. Previous survey-based studies have reported that dislike for school food and peer-pressure are key predictors for school lunch skipping, in Sweden and abroad [11, 13]. Furthermore, the food environment surrounding the school may influence the student’s eating behaviours, and greater exposure to retailers selling energy-dense, nutrient-poor foods has been associated with less healthy food choices [14, 15]. For example, an increased density of fast-food outlets and grocery stores in the school neighbourhood has been associated with higher self-rated fast-food consumption, school lunch skipping and snacking outside of schools [12, 16].

Nevertheless, exploring adolescents’ dietary patterns within school food environments presents several methodological challenges. Previous research has largely relied on self-reported measures such as surveys and food diaries, which are prone to recall and social desirability bias [11–14, 17]. Reviews have highlighted additional limitations, including diverse and imprecise measures of the food environment, often based on assumed rather than observed student exposures to food retailers and purchasing behaviours [10, 16, 18]. This underscores the need for more objective approaches when exploring dietary patterns within school food environments.

The need is particularly evident in Sweden, where the context differs from that of many previously studied countries, as all compulsory schools (ages 7–16) provide free, nutritious lunches, and most upper secondary schools (ages 16–19) do so as well, although this is not legally required [19, 20]. In addition, Swedish schools establish their own regulations regarding whether students are permitted to leave school premises during breaks [19]. A previous survey has shown that the majority of lower- and upper secondary schools (70% and 98%, respectively) allow students to leave the premises freely during the school day [21]. This creates conditions in which nearby food retailers may strongly influence students’ eating behaviours. Despite this, limited information is available on the extent to which Swedish students skip school lunch and what they consume instead.

This study aims to investigate the dietary patterns among adolescent students during school hours in central Stockholm, Sweden, focusing on eating patterns associated with school lunch participation and snacking in and around school. To achieve this aim, a novel smartphone-based monitoring methodology is employed in which students act as citizen scientists to collect pictures of their meals and beverages. By integrating image-based dietary reporting with information on the timing and location of eating occasions, the approach enables assessment of both the nutritional quality and the contextual setting of meals consumed within and around school, providing richer insights than traditional survey-based methods.

## Method

### Study design

This cross-sectional study examined dietary patterns during school hours in central Stockholm and pooled data from two studies gathered at several time-points with independent student samples over the course of four years. The aim of the study was to investigate lunch patterns and snacking behaviours inside and around school among adolescent students. For the purpose of this study, analyses focused on eating occasions occurring during weekdays between 07.30 and 17:00, selected to capture meals and snacks consumed during the school day.

Data were collected within the European project *Big Data against Childhood Obesity* (BigO) [22] in spring and autumn 2019, autumn 2020 and spring 2021, and within the FORTE-funded *AdSafe* project in spring 2024. The Covid-19 pandemic emerged in spring 2020, leading to a three-month closure of upper secondary schools in Sweden [23]. However, data collection resumed as planned in autumn 2020 after schools reopened. Data collection included self-reported images of students’ meals and beverages, complemented by a questionnaire on dietary habits. The study size was determined by the availability of data from the two pooled studies and included all participants contributing data during the respective data collection periods.

### Participants and recruitment

Participants for the study were students aged 13–19 years, recruited from a lower and an upper secondary school in central Stockholm, with approximately 700 and 550 students, respectively. Both schools have an international profile (i.e., part of the curriculum is taught in English) and are situated in the southern part of central Stockholm. The upper secondary school took part in both data collection efforts in 2019–2021 and 2024, while the lower secondary school only took part in the second data collection in 2024. The participating classes were selected by the schools themselves, enrolling students who provided informed consent. Only participants who contributed at least one valid meal or beverage image were included in the analytical dataset and are represented in the results.

### Smartphone application

**Fig. 1:**
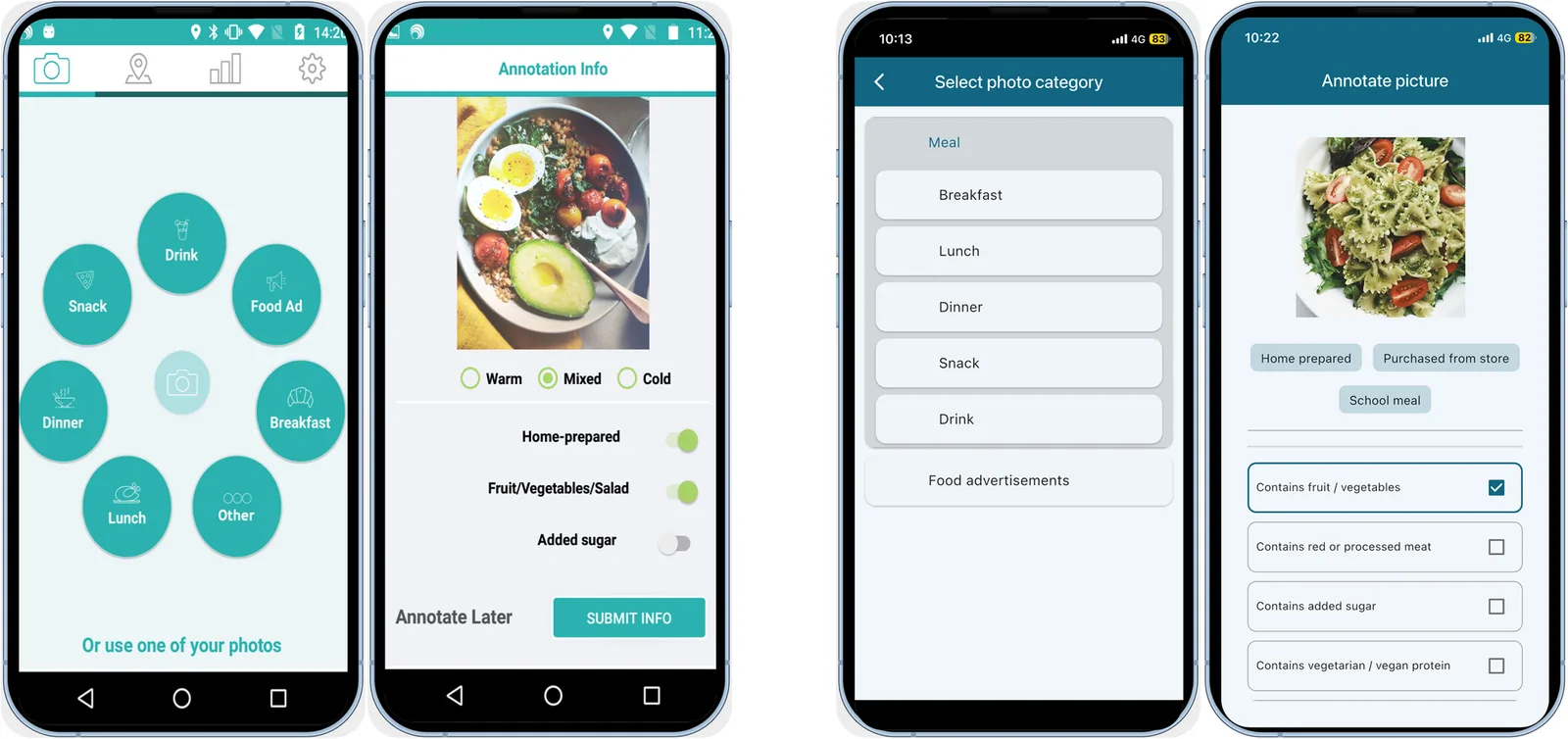
Data collection apps; on the left, “myBigO”, on the right, “Mobile Sense”

Two smartphone apps were used for the data collection for the two study periods (Fig. 1). In the first period, a research app (i.e., BigO app) was developed specifically for the project [24]. For the 2024 data collection, a new research app (Mobile Sense; Arbisense AB; Stockholm; Sweden) was developed with similar functionality [25], as the BigO project had concluded in 2021 and the BigO app was no longer available. Both apps were free to download from App Store and Google Play Store, but access was limited to participants via study-specific passwords.

In both apps, participants could take and upload images of their meals and beverages. To upload an image, participants selected the meal type (breakfast, lunch, dinner, snack, drink) and took a photo through the app. In doing so, the app automatically also recorded both the location of the event (GPS coordinates) and the time it took place. Participants then had the option to provide annotations describing the content of the picture (e.g., fruit and vegetables, red and processed meat, added sugar). Both apps provided visual feedback to the participants about their data contribution, supporting their role as citizen scientists. Daily notifications were used to help participants remember to contribute data.

### Data collection

Data collection started with researchers visiting the participating classes, instructing students on how to download and use the app. During this session, an onboarding questionnaire was answered to collect participant characteristics. In the second round of data collection in 2024, the participants also answered a 14 item-questionnaire about their dietary habits (Additional file 1: Table S1). Afterwards, the participants were asked to upload pictures of all their meals and beverages as they happened for two consecutive weeks, including meals that were eaten at school. The data collection was uncontrolled, with no requirements for picture contribution, aiming to capture the participants’ actual dietary habits. All the submitted data were manually reviewed by researchers to ensure only relevant images for the study were collected.

### Image annotation

For the analysis of all the collected data, two independent annotators (both trained nutritionists) manually annotated the images, using common predefined annotation schemes. The annotation schemes were similar to those used in previous studies [26], but were adapted for the specific study through in-house consensus meetings with the participation of seven independent scientists (including dieticians, nutritionists, behavioural and clinical scientists, and research engineers). This process resulted in the creation of three schemes, that were used for the content annotation of all the collected pictures. As individual images could contain both meals and beverages, some images were included in both the meal and beverage annotation datasets. Consequently, the combined number of meal and beverage observations exceeds the total number of unique images.

The first scheme classified images containing food by meal type (e.g., sweet snack, nutritious meal/snack, junk food). “Junk food” referred to foods typically high in saturated fat and salt, such as fast-food. The category “other unhealthy foods” included foods that were not classified as junk food but were considered inconsistent with dietary recommendations due to their composition, such as meals high in processed meat, saturated fat and/or salt. “Nutritious meal/snacks” were defined as complete meals or snacks broadly consistent with dietary recommendations. The category “other” included food items that were generally consistent with dietary recommendations but did not constitute a sufficiently complete meal or snack to be classified as a nutritious meal/snack (e.g., single food items such as bread or potatoes). The distinction between categories was not always clear-cut and should be viewed as a continuum rather than as strictly separate groups. To improve interpretability and facilitate statistical analyses, the detailed categories were aggregated into broader healthy and unhealthy food groups rather than analysed as separate categories. The categories “junk food”, “other unhealthy foods”, “sweet snack” and “savoury snack” were combined into a composite variable labelled as *Unhealthy Foods*, based on the World Health Organization’s definition of foods rich in salt, free sugar, saturated fats and trans-fatty acids [27]. Similarly, the categories “nutritious meal/snack” and “other” were combined into a variable labelled as *Healthy Foods*, defined as foods broadly consistent with the Nordic Nutrition Recommendations [4]. As “other” included food items generally consistent with dietary recommendations, this category was included within the healthy food group. Taken together, these two variables represented the total picture dataset.

The second scheme detailed specifics about the meal, indicating whether it contained substantial amounts of e.g., fruits, vegetables, red and processed meat. The third scheme focused on beverages, specifying drink type and potential sweetener. Afterwards, the schemes were finalized based on consensus discussions on observed annotation challenges, informed by the annotators’ work on a separate dataset of 1000 similar images collected through independent but comparable studies. The finalized schemes are presented in Additional file 2: Figure S1.

**Fig. 2:**
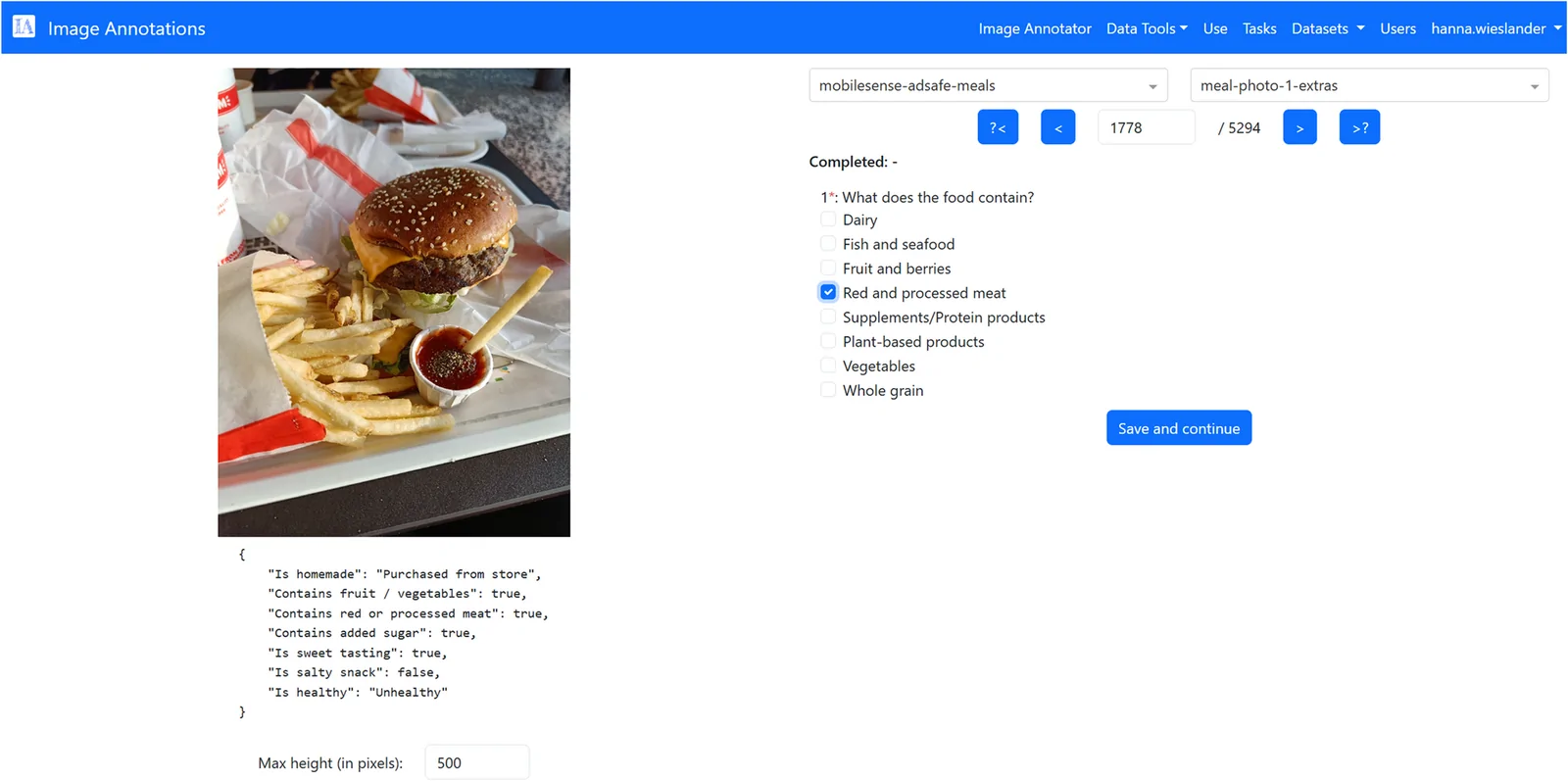
Annotation tool illustrating the second scheme used to classify specifics of the meal

The final experimental picture dataset was divided equally between the two annotators and each image was annotated by one annotator only, using a dedicated annotation software tool developed within the research group (Fig. 2). To support the annotators during the process, any in-app annotations provided by participants were displayed beneath each image within the tool. In addition to analysing the study dataset, inter-rater agreement was evaluated on a separate agreement dataset of 500 images from similar previous studies, which was independently annotated by both annotators and used to calculate Cohen’s Kappa (K). The dataset consisted of comparable meal and beverage images collected from students during school hours and was considered sufficiently similar to the study dataset to provide an indication of annotation consistency. K was interpreted according to the following guidelines: No agreement < 0.21, Minimal agreement 0.21–0.39, Weak agreement 0.40–0.59, Moderate agreement 0.60–0.79, Strong agreement 0.80–0.90, and Almost perfect agreement > 0.90 [28].

### Statistical analysis

The annotations were exported from the annotation software tool to a CSV file, for further analysis. The data was filtered on GPS and timestamps, to only include images taken during weekdays between 07:30–17:00. Additionally, based on the GPS tagging, only pictures in and around the two schools were considered in a radius of approximately 1.5 km. All statistical analyses were conducted in R version 4.4.2 (R Core Team, 2024).

Pearsons’s chi-square test was used to compare the proportion of unhealthy food reported inside vs. outside of school in the two study-period datasets, analysed separately and combined. However, since each participant could contribute multiple images, image-level observations are not necessarily independent. Thus, a sensitivity analysis was performed using a logistic mixed-effects models with participant as a random intercept to account for within-participant correlation. These analyses generally supported the findings obtained using Pearson’s chi-square test, and are presented in Additional file 1, Table S2. As the main patterns were consistent across analyses, only results from the combined dataset are presented in the result section. Details of the separate analyses and comparisons between study periods are provided in Additional file 1: Table S3 and Table S4. Additional analyses on the combined dataset examined specific meal and beverage components inside vs. outside of school. For expected cell counts < 5, Fisher’s exact test was used instead. Adjusted or subgroup analyses were not performed, as individual-level covariates collected through the onboarding questionnaire could not be linked to individual images. A significance level of 0.05 was applied to all tests.

### Exploratory bias analysis

Finally, the distributions of the contribution patterns among participating students were examined, taking into consideration the number of images uploaded by each user during the two-week data collection period. Two independent analyses were performed, one excluding the extreme contributors, defined as the ten participants with the highest number of submitted images, and another excluding minimal contributors, defined as participants who contributed < 2 pictures each. In both cases, all food and beverage category comparisons were repeated to evaluate the influence of extreme and minimal contributors on the reported study outcomes. Note that, given the highly skewed nature of the data, data are presented using a logarithmic scale to highlight differences across levels of participation.

### Ethical considerations and data management

This study was approved by the Swedish Ethical Review Authority (BigO Dnr: 2018/1921-31/5 and AdSafe Dnr 2023-01085-01). Prior to participation, information meetings were held with all included classes to provide detailed information about the study and allow students to ask questions. During the meetings, students received information letters about the study along with consent forms. For students under 15 years of age, a legal guardian’s signature was also required. All students in the selected classes who returned signed consent forms were included in the study. Participation was entirely voluntary, and participants could withdraw at any time, in which case all associated data were permanently deleted. After completion of the study, participants received a cinema ticket as a compensation for their participation, regardless of the amount of data contributed.

The apps used in the study assigned random usernames to the participants, ensuring pseudonymization of all data. A key file linking usernames to participants’ names was stored separately from all other study data and was accessed only by authorized researchers if inappropriate data were uploaded or technical issues required contacting the participant (this was eventually not necessary in any of the data collection periods). Images and in-app questionnaire responses were uploaded to secure servers, and images were displayed in a cloud-based dashboard accessible only to authorized researchers, where they were reviewed. Any irrelevant image to the study (i.e., not a meal or beverage image) was manually deleted by the researchers. The 14-item food questionnaire in the 2024 study period was completed anonymously via REDCap, a secure and web-based survey and data management tool [29], and responses were stored on local servers at Karolinska Institutet.

## Results

### Participant characteristics

In total, 483 students participated in the study, out of which 314 participated in the first (2019–2021), and 169 participated in the second (2024) study periods. Participant and school characteristics, and data contributions are presented in Table 1.

**Table 1:** Participant and school characteristics and data contributions

Category	School 1 (SP1^b^)	School 1 (SP2)	School 2 (SP2)	Combined
Participant characteristics				
Participants (n)	314	89	80	
Age, years (mean ± SD)	15.9 ± 0.8	16.5 **±** 1.0	14.9 **±** 1.3	16.0 ± 1.0
Gender (%)				
Female	63.1	58.8	56.7	61.6
Male	35.5	36.0	33.3	35.5
Other/Don’t want to answer	1.4	5.2	10.0	2.9
School characteristics				
School café	No	No	Yes	**–**
Vending machines	No	No	No	**–**
Lunch provision^a^	External provider	External provider	Nearby school	**–**
Data contributions				
Meal contribution (n)	2323	588	459	3370
Lunch	1940	516	285	2741
Snack	383	72	174	629
Beverage contribution (n)	1038	296	153	1487

^a^ School lunches are provided to both schools through catering

^b^ SP = study period (SP1 = 2019–2021; SP2 = 2024)

### Inter-rater agreement analysis

A strong agreement was observed between the two annotators for the main categories “Unhealthy” and “Healthy” (K = 0.82 for both). For the majority of the beverage sweetener annotations, agreement ranged from strong to almost perfect (K = 0.85–0.94). Exceptions included the “not sure” category (K = 0.46), which had few observations. Despite this, a high percentage agreement (99%) was still observed. For the food type categories, agreement ranged from moderate to almost perfect (K = 0.70-1.00).

### Self-rated food questionnaire

The 14-item questionnaire, completed by participants in the second study period in 2024, revealed that almost 50% of participants reported eating school lunch every day. In addition, 69% of the participants reported leaving school to buy something to eat at least once per week. While Table 2 presents the questions on self-reported frequency of consumption of selected food items, questions 11, 12 and 14 addressed other aspects and are summarized in the text below. The full distribution of responses for these questions is presented in Additional file 1: Table S5.

**Table 2:** Frequency questions from the 14-item questionnaire

Question^a^	≥ 3/day^b^ *n* (%)	2/day *n* (%)	1/day *n* (%)	5–6/week *n* (%)	3–4/week *n* (%)	1–2/week *n* (%)	< 1/week or never *n* (%)
Q1: Fast-food meal (*n* = 167)	9 (5.4)	5 (3.0)	9 (5.4)	5 (3.0)	28 (16.8)	65 (38.9)	46 (27.5)
Q2: Sweets and chips snack (*n* = 166)	3 (1.8)	12 (7.2)	11 (6.6)	20 (12.1)	60 (36.2)	46 (27.7)	14 (8.4)
Q3: Vegetable intake (*n* = 166)	19 (11.4)	54 (32.5)	34 (20.5)	22 (13.3)	27 (16.3)	8 (4.8)	2 (1.2)
Q4: Fruit intake (*n* = 166)	11 (6.6)	25 (15.0)	33 (19.9)	23 (13.9)	39 (23.5)	29 (17.5)	6 (3.6)
Q8: Processed meat intake (*n* = 166)	5 (3.0)	11 (6.6)	5 (3.0)	23 (13.9)	41 (24.7)	46 (27.7)	35 (21.1)
Q9: Whole-grain intake (*n* = 167)	10 (6.0)	20 (12.0)	22 (13.2)	25 (15.0)	33 (19.7)	43 (25.7)	14 (8.4)

	≥ 4/week *n* (%)	2–3/week *n* (%)	1/week *n* (%)	< 1/week *n* (%)	Never *n* (%)		
Q5: Sugary-drink intake (*n* = 166)	18 (10.8)	35 (21.1)	26 (15.7)	45 (27.1)	42 (25.3)		
Q6: Light drink intake (*n* = 167)	12 (7.2)	36 (21.6)	27 (16.2)	38 (22.7)	54 (32.3)		
Q7: Fish intake (*n* = 167)	3 (1.8)	57 (34.1)	46 (27.5)	38 (22.8)	23 (13.8)		

	0 days *n* (%)	1 day *n* (%)	2 days *n* (%)	3 days *n* (%)	4 days *n* (%)	5 days *n* (%)	
Q10: School lunch frequency (*n* = 167)	6 (3.6)	4 (2.4)	8 (4.8)	21 (12.6)	45 (26.9)	83 (49.7)	
Q13: Meal outside school frequency (*n* = 167)	52 (31.1)	58 (34.7)	27 (16.2)	24 (14.4)	5 (3.0)	1 (0.6)	

^a^ Codes correspond to questionnaire items, full questions are provided in Additional file 1: Table S1

^b^ Results are presented as percentages of students answering each question, varying n is shown in the table

Responses to the questionnaire showed that 44.3% of participants reported some level of dissatisfaction with the school lunch, while 32.0% reported some level of satisfaction (Additional file 1: Table S5). Among participants who did not always eat school lunch, most reported skipping lunch entirely on those days (30.2%), followed by purchasing food from a fast-food restaurant (25.3%) or a grocery store (21.6%). Although “none of the above” was a common response, the most frequently reported food categories bought outside school was “candy, ice cream, cookie or other pastries” (38.2%), followed by “energy drinks” (29.3%) and “fast-food” (24.2%).

### Meal and beverage reporting during school hours

**Fig. 3:**
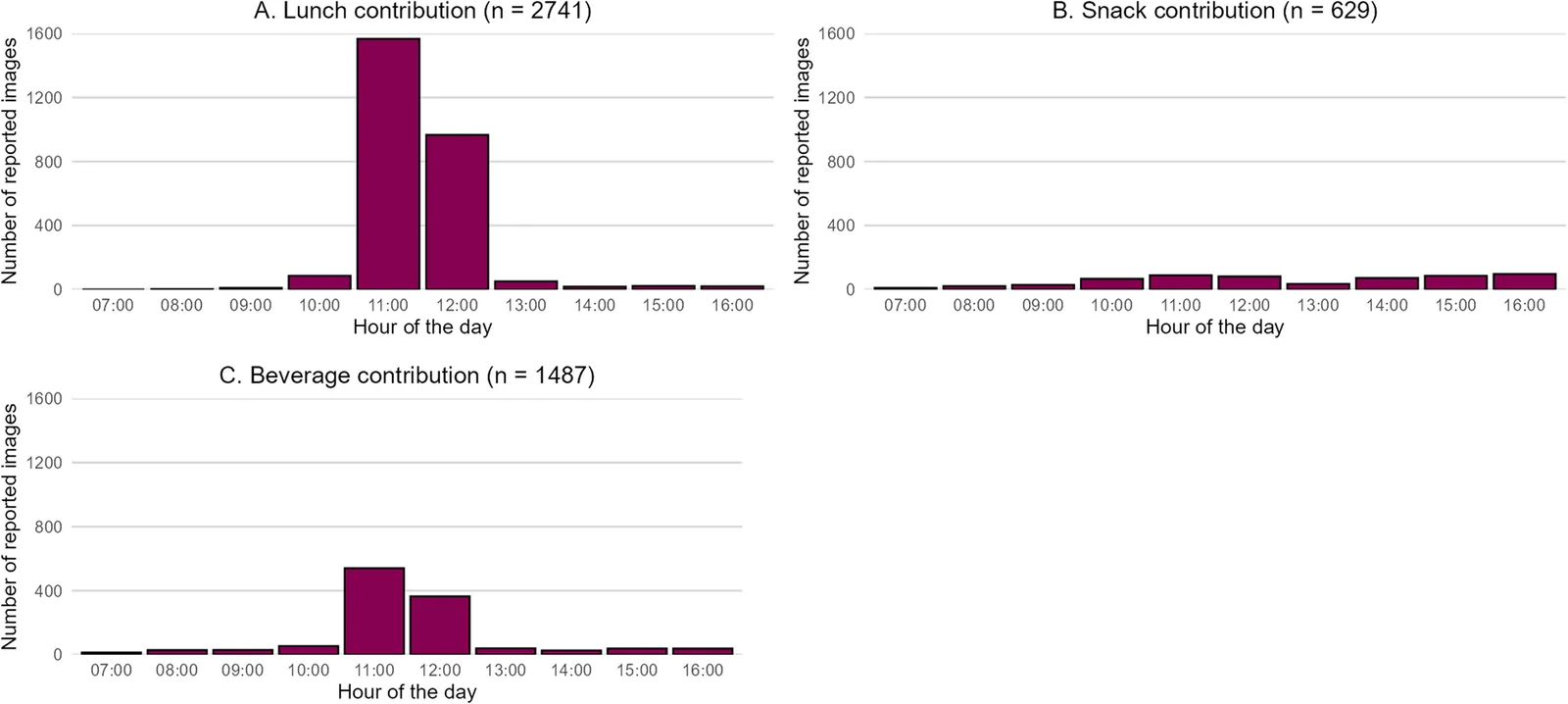
Hourly image contributions during school hours for **A** lunches, **B** snacks, **C** beverages

Participants reported most images of lunches and beverages between 11:00 and 12:00 (Fig. 3). Snacks were reported continuously throughout the day, with one peak around lunchtime, and one in the afternoon.

### Meal location and nutritional patterns

**Fig. 4:**
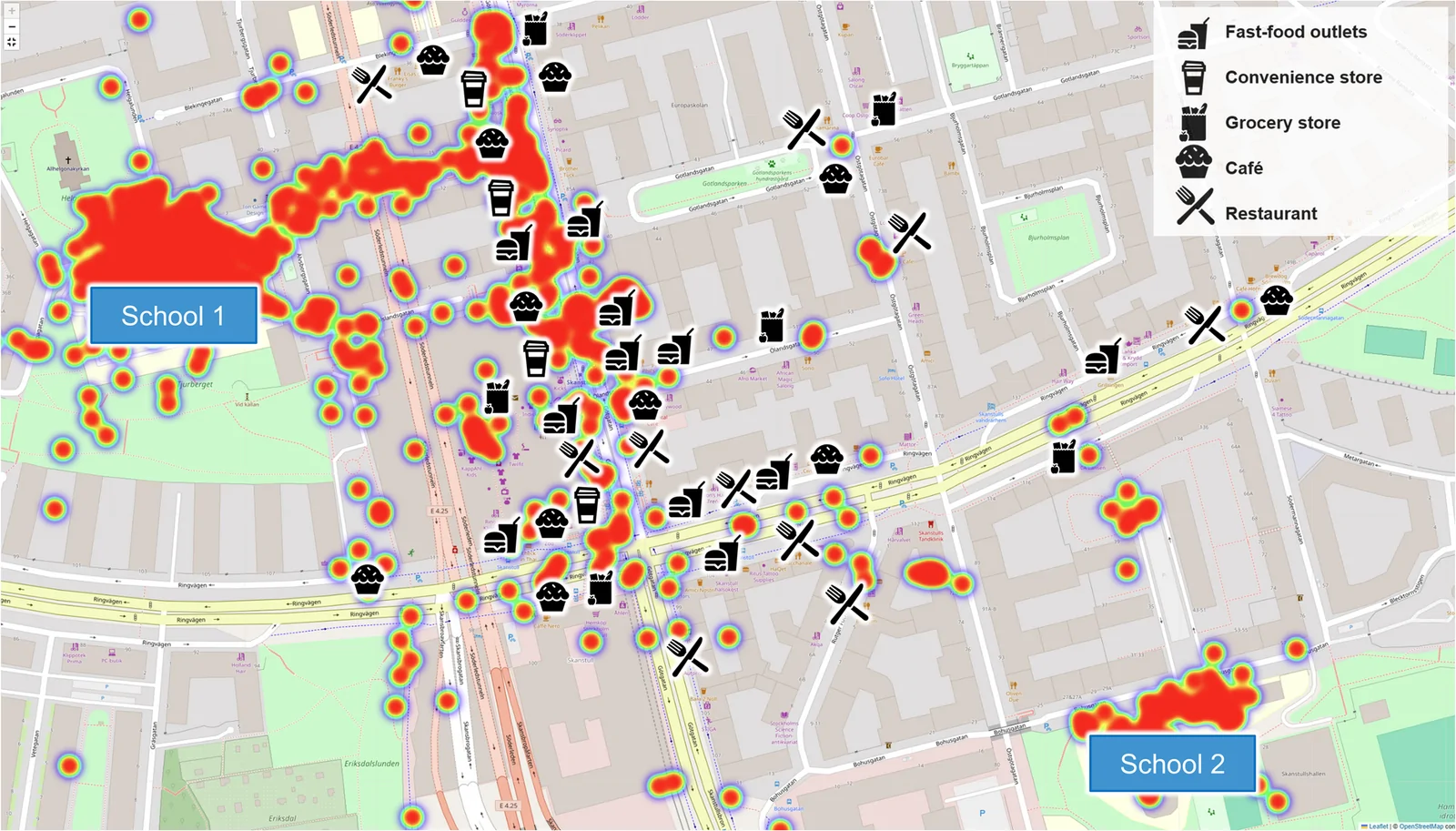
Map of reported image locations, highlighting food retailers identified based on the images

The most frequent eating locations are visualized in Fig. 4, based on the GPS tags of the collected pictures. Each red point represents a location where at least one meal or beverage image was taken, with larger red areas indicating locations with a higher density of reported images. The map also indicates food retailers identified post-data collection, using public datasets (e.g., OpenStreetMaps) through the reported images, including fast-food outlets, convenience stores, grocery stores, cafés and restaurants.

**Fig. 5:**
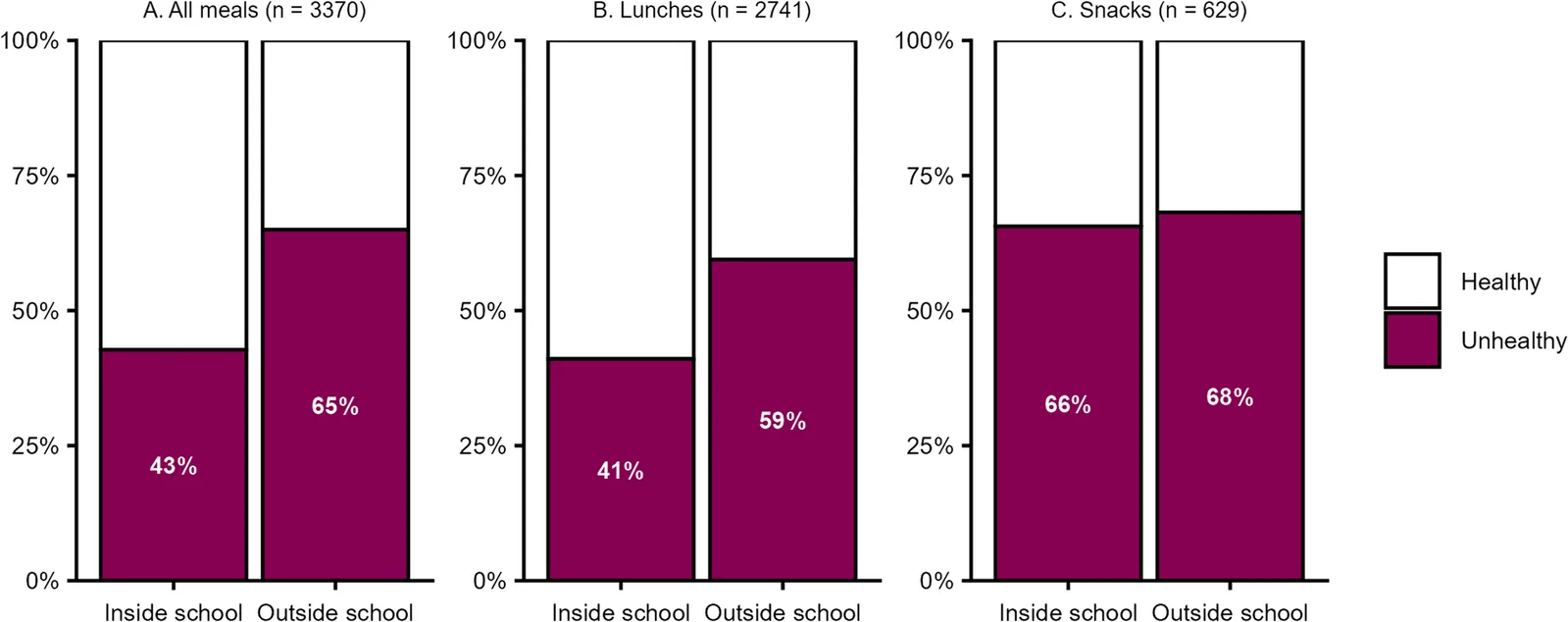
Proportion of healthy vs. unhealthy meals: lunches and snacks combined (**A**), lunches (**B**), snacks (**C**)

Out of all meals eaten during school hours, 21% were eaten outside of school premises. Most meals eaten outside school were categorised as unhealthy (Fig. 5). The proportion of unhealthy meals was significantly higher outside school than inside (65% vs. 43%; χ^2^ = 109.6, *p* < 0.001).

Inside school students reported almost exclusively lunches (93%), compared to outside school where snacks were more prevalent (64%). The proportion of healthy lunches were significantly higher inside school (59% inside vs. 41% outside; χ^2^ = 31.9, *p* < 0.001, Fig. 5). The majority of snacks were classified as unhealthy, and no significant difference was observed for snacks between the two locations (χ^2^ = 0.4, *p* = 0.529).

The corresponding logistic mixed-effects sensitivity analysis, accounting for within-participant correlations, produced broadly similar findings (Additional file 1, Table S2). For all meals, the association between eating location and unhealthy food remained significant (OR 3.36, 95% CI 2.71–4.16, *p* < 0.001). Similar patterns were observed for lunches and snacks. While the overall conclusion remained unchanged, the snack-specific analysis reached statistical significance in the mixed-effects model whereas the primary chi-square analysis did not.

**Fig. 6:**
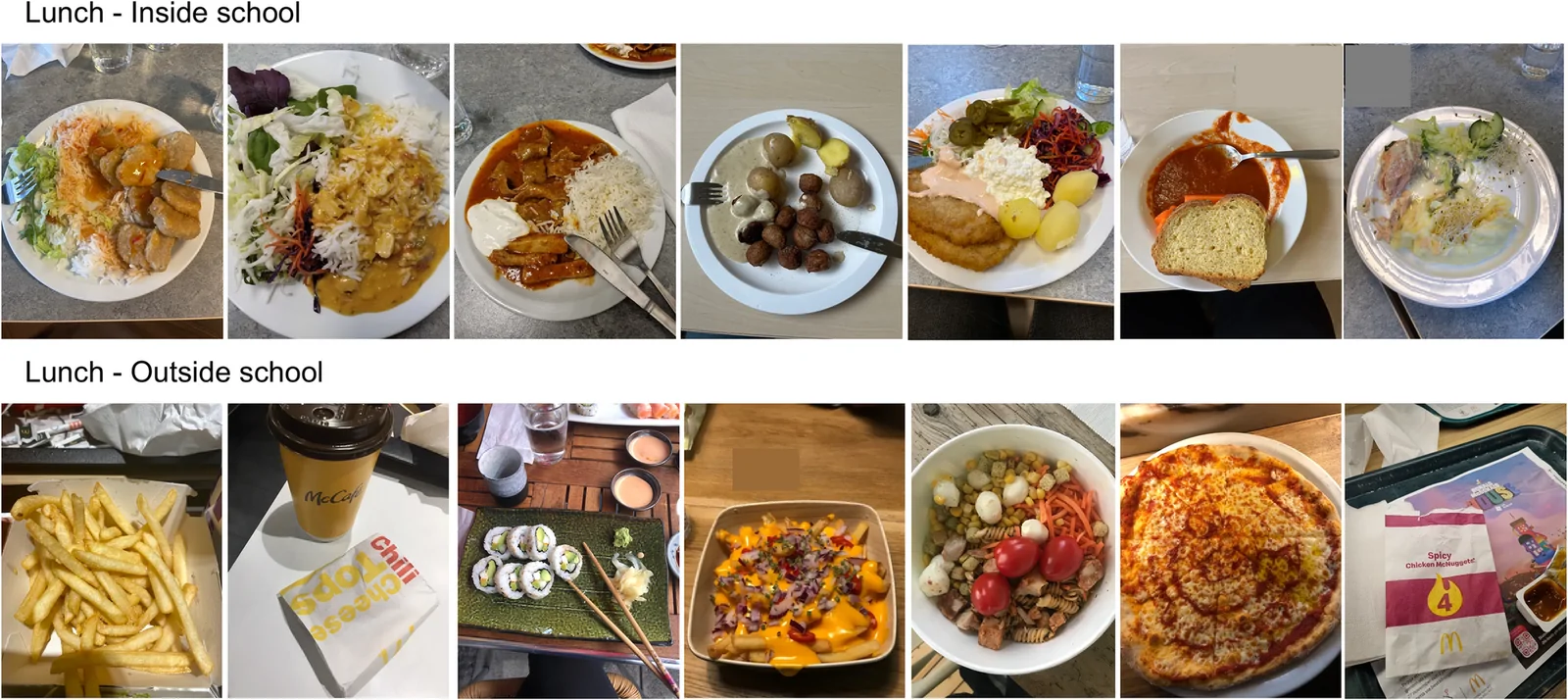
Selection of lunch-images uploaded inside- (top row) and outside school (bottom row)

The meals eaten inside of school contained significantly higher percentages of vegetables, red and processed meat, and fish and seafood, compared to outside of school. For instance, vegetables were included in 38% of meals consumed inside school vs. 13% outside school (χ^2^ = 149.1, *p* < 0.001); red and processed meat in 38% vs. 19% (χ^2^ = 91.4, *p* < 0.001); and fish and seafood appeared in 9% vs. 2% (χ^2^ = 39.5, *p* < 0.001). On the other hand, meals eaten outside of school contained higher percentages of fruit and berries (1% inside vs. 9% outside, χ^2^ = 114.4, *p* < 0.001) and whole grains (3% inside vs. 5% outside, χ^2^ = 10.4, *p* = 0.001). No significant difference was observed for dairy between the two locations (6% inside vs. 8% outside, χ^2^ = 2.9, *p* = 0.089). Plant-based products and supplements/protein products occurred too infrequently in the reported images and were therefore not included in the comparative statistical analyses or result presentation. Figure 6 provides illustrative examples of lunch images inside and outside school, highlighting variation in meal composition between the two settings.

### Beverages

Of the 1487 images containing beverages, 24% were reported outside school. Sweetened beverages were more frequently reported outside of school than inside. Specifically, sugar-sweetened beverages accounted for 32% of beverages outside school compared to 7% inside (χ^2^ = 158.5, *p* < 0.001). Artificially sweetened beverages were found in 6% of beverages outside vs. 1% inside (χ^2^ = 48.3, *p* < 0.001). Non-sweetened beverages were the most reported in both settings, but significantly more common inside the school than outside (93% vs. 49%, χ^2^ = 346.8, *p* < 0.001). Notably, 80% of the drinks reported inside the school was water.

### Exploratory bias analysis

**Fig. 7:**
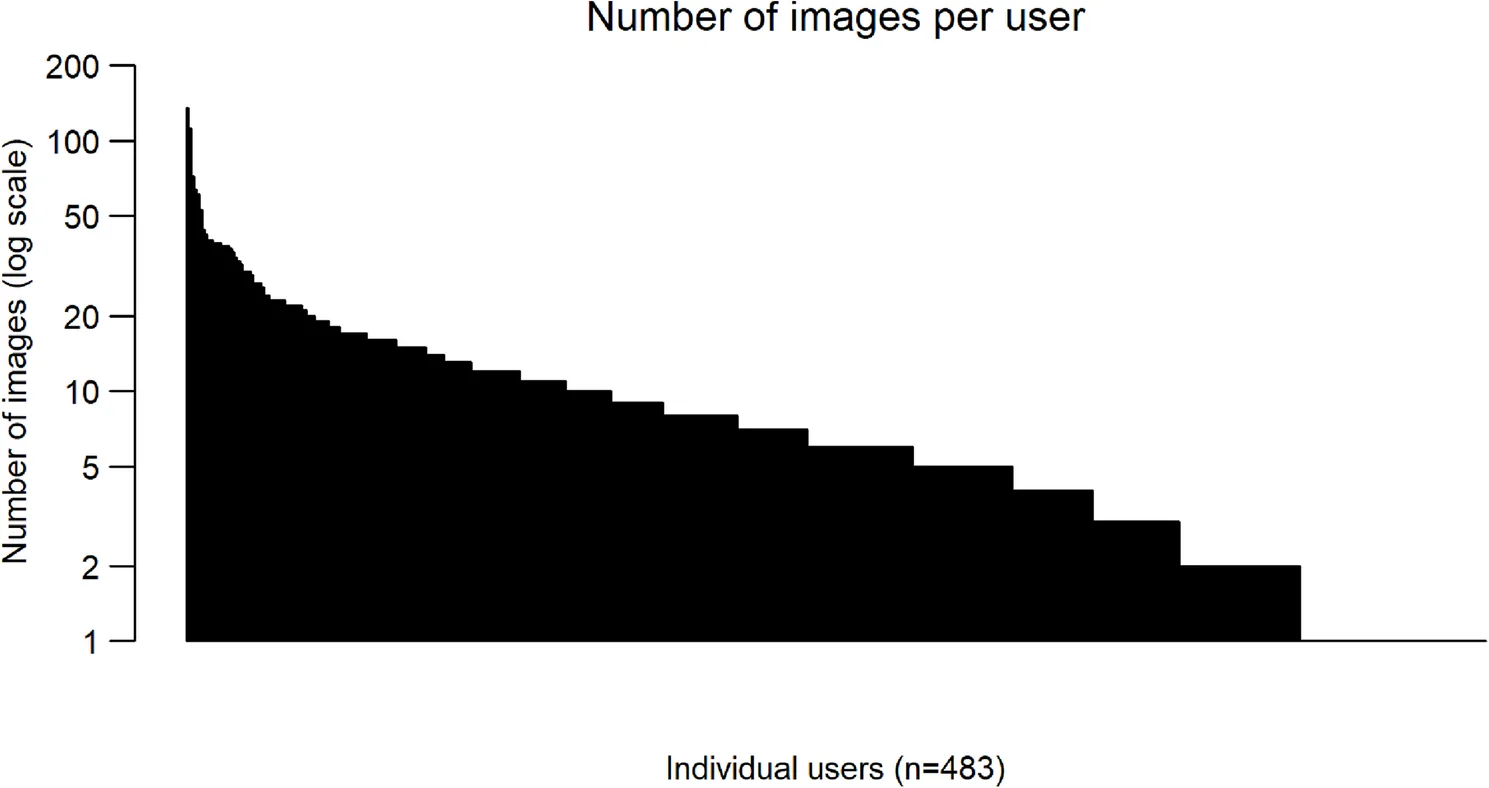
Distribution plot of the contribution patterns observed among the participating students

Figure 7 illustrates the distributions of the contribution patterns among all 483 participating students. The highest individual contribution was 135 images, while 69 participants reported one image each.

Eight participants contributed beverage images only and were therefore not included in the meal-image sensitivity analyses. Among the 475 participants contributing at least one meal image, exclusion of the ten highest contributors resulted in a dataset comprising 465 participants and 2944 images. The proportion of unhealthy meals remained significantly higher outside school compared to inside (χ^2^ = 107.1, *p* < 0.001). After excluding participants contributing < 2 meal images, the analysis included 404 participants and 3299 images, and a similar pattern was observed (χ^2^ = 101.3, *p* < 0.001). Results were consistent across analyses of food type and beverage sweetener, where categories that were statistically significant in the full dataset remain significant, while non-significant categories continued to be non-significant (see Additional file 1: Table S6, for all test statistics). Characteristics of the datasets used in the exploratory bias analysis are presented in Additional file 1: Table S7. The exclusions altered the sample sizes as expected but resulted in only minor changes to the distribution of observations across study periods.

## Discussion

### Result discussion

This study used a smartphone-based approach to examine dietary patterns among adolescents in central Stockholm, focusing on students’ habit to exit the school grounds to eat lunch in the surrounding area, a habit that had long been identified but not previously quantified. Meals consumed inside of school were generally healthier and contained significantly higher proportions of vegetables, red and processed meat, and fish and seafood compared with those eaten outside of school grounds. This pattern likely reflects the fact that school-provided meals are served as a buffet with multiple components, enabling students to assemble a more complete meal that typically includes a source of carbohydrate, protein, and vegetables. In contrast, lunches consumed outside school tended to be more snack-based, even when reported as lunches, as illustrated by the example images presented in Fig. 6.

It should be noted that the image-based methodology captured reported meals rather than actual consumption. Therefore, although the images provide valuable information about food choices and eating locations, they cannot determine whether the photographed foods were consumed in full, partially consumed, shared with others, or discarded. Similarly, the images do not reveal whether meals consumed outside school replaced the school-provided lunch, supplemented it, or represented an additional eating occasion.

### Comparison with previous research

This is the first study to report out-of-school meals using meal pictures, finding that 21% of all reported meals during school hours were consumed outside of school. Questionnaire data from this study provide additional context to these findings. Approximately 77% of respondents reported eating the school-provided lunch on at least four days per week. Although this suggests that overall school lunch participation was relatively high, only half of the respondents reported eating the school-provided lunch every school day. Previous Swedish research has shown that school lunch participation tends to decline with age [13]. Among younger Swedish children aged 8–11 years, only 12% reported eating school lunch less than five times per week [13], compared with approximately 50% of adolescents in the present study. However, direct comparisons should be interpreted with caution, as the previous study did not investigate whether students left school premises or what foods were consumed when school lunch was not eaten.

Several factors have previously been associated with school lunch skipping, including dissatisfaction with the school lunch, long cafeteria lines, noisy or stressful dining environments and the perception that food available outside school is more appealing and offers greater variety [11, 30, 31]. Dissatisfaction with the school lunch also tends to increase with age [32]. In the present study, almost half of the questionnaire respondents reported being dissatisfied with the school lunch. Combined with school-level regulations that commonly allow students in the upper grades to leave school premises during the day [21], these factors may contribute to lower school lunch participation among adolescents. Taken together, the image data suggest that eating outside school during school hours is common, while the questionnaire findings indicate that school lunch skipping may occur among a proportion of students. These findings highlight the need for strategies to improve satisfaction with the free school lunch and to encourage students to eat the provided meal.

The lunches reported outside of school were generally less healthy than those reported inside. Spatial data linked to the meal pictures revealed that *fast-food outlets*, *cafés*, *convenience stores*, *restaurants* and *grocery stores* located near the two centrally situated schools were common sources of the reported meals and beverages. This aligns with previous research showing that unhealthy food environments surrounding schools are associated with higher fast-food consumption [12, 33, 34], although national variation has been observed depending on school policies and meal systems [35]. In the Swedish context, the present findings indicate that adolescents frequently buy food from nearby retailers during the school day. In addition to easy access to food retailers, previous research has documented high exposure to unhealthy food marketing among schoolchildren in central Stockholm, often involving price promotions [36]. As such marketing has been linked to increased preference, purchasing and intake of unhealthy foods [37], this illustrates a school food environment that tend to steer students towards less healthy options. Given that more than half of Swedish lower and upper secondary schools have two or more food vendors within 500 m [21], these findings highlight a broader public health concern, as the current food environment provides easy access to unhealthy alternatives and does not sufficiently support healthy dietary habits among students.

Although meals consumed inside school were generally healthier than those eaten outside, this study also revealed challenges related to the school lunches. In Sweden, school lunches contribute significantly to students’ overall diet, including an important share of their daily vegetable intake [9]. Consistent with previous research, this study observed a higher proportion of healthy lunches and vegetables in meals reported inside school. However, 41% of all school lunches were still classified as unhealthy, largely due to high levels of processed meat products such as kebabs, sausages and nuggets. A previous mapping of Swedish school lunches has shown similar patterns, with several schools serving red and processed meat as often as four to five days per week [21]. Since processed meat is a major contributor to the diet-related disease burden in Nordic countries [4, 38], these findings highlight the need to reduce exposure in settings where meals are publicly provided. Efforts should therefore focus not only on encouraging students to eat at school, but also on improving the healthiness of the meals served, particularly by limiting the amount of processed meat.

In addition to lunches, the reported snack images were generally unhealthy both inside and outside of school grounds. This aligns with questionnaire data indicating that students often purchase sweet snacks, energy drinks and fast-food when outside the school grounds. Most snack consumption was reported at lunchtime and in the afternoon, suggesting that some snacks may have replaced or complemented the school lunch. It should also be noted that the study captured where foods were consumed rather than where they were purchased. Therefore, snacks consumed inside school may have been brought from home or purchased elsewhere before consumption. Given the limited availability of food outlets within the participating schools, with only one of the schools having a cafeteria and neither school having vending machines, unhealthy snacks reported at school do not necessarily reflect purchases made in the school environment itself. Instead, they may partly reflect exposures to the broader food environment surrounding the school. Previous research has shown that proximity to fast-food outlets and grocery stores is associated with irregular eating habits, including school lunch skipping and purchasing snacks outside of school [12]. It is also linked to afternoon snacking and snacking during school commutes [18, 39], which is reflected in the afternoon peak in snack reporting in this study. These findings underscore the need to develop strategies that promote healthier snacking during school hours and create supportive food environments both inside school and in the nearby environment.

### Strengths and limitations

A key strength of the study is the detailed, annotated data collected through photographs linked to both time and GPS coordinates, which enabled precise information about when and where meals were consumed. Using images to collect data has previously proven effective in research with younger populations, as it is accessible, user-friendly, and facilitates the collection of contextual details that are often difficult to recall through self-report, such as exact meal times and locations [40, 41]. By reducing participant burden and removing the need for students to categorize foods themselves, this method also lowers the risk of recall bias, a common challenge with self-reported dietary data [17]. Instead, trained nutritionists classified all meals and beverages, ensuring accurate and consistent categorization, supported by high inter-rater agreement.

Unlike previous studies that have struggled to accurately assess exposure to food retailers [18], this approach focuses on the actual places students visit when eating, providing a more accurate mapping of the school food environment. The combination of time-stamped and spatial data not only provides unique insights into students’ dietary patterns but also holds considerable potential for larger-scale applications. At the population level, such data can support precise mapping of eating locations, detailed characterization of food environment exposures, and the development and evaluation of nutrition-related interventions across different settings.

Despite these strengths, several limitations should be acknowledged. First, the method relies on participant compliance, which allows for selective reporting and missing data when meals or beverages are not photographed. This was shown in the exploratory bias analysis, where 69 participants submitted only one image. Participation levels also varied substantially between students, and the primary analyses were performed at the image level. Consequently, observations contributed by the same participant were not fully independent, and participants contributing many images may have had a greater influence on the observed patterns than participants contributing only a few images. However, supplementary mixed-effects analyses accounting for participant-level clustering produced results that were broadly consistent with the primary analyses, suggesting that the overall findings were robust to within-participant dependence. The only notable difference was observed for snacks, where the mixed-effects model identified a statistically significant association that confirmed the non-significant trend that was observed in the primary chi-square analysis. However, this did not substantially alter the overall interpretation of the findings, as unhealthy snack consumption was common both inside and outside school (66% vs. 68%), despite the difference in statistical significance between analytical approaches. In addition, exploratory bias analyses excluding both extreme and minimal contributors also supported the findings of the primary analyses (Additional file 1: Table S3). Nevertheless, unequal participation levels may have affected the representativeness of the dataset, and the findings should primarily be interpreted as patterns observed in the submitted image dataset rather than precise estimates of the prevalence of dietary behaviours among students.

Secondly, although the combined dataset from the first and second study periods was used for the main analyses, some differences between the datasets were observed (Additional file 1: Table S4). These findings suggest that temporal, school-level, or cohort-related differences may have influenced the observed proportions. However, the key patterns of interest, including less healthy lunches outside school and frequent consumption of unhealthy snacks, were consistent across study periods, supporting the decision to pool the datasets. The repeated cross-sectional design, combined with the inability to link age, gender and school characteristics to individual images, also limited opportunities for adjusted analyses. As a result, observed differences in meal location and dietary quality may partly reflect age-related dietary behaviours, gender-related food choices, school-specific characteristics, or differences in the characteristics of participants across study periods. Consequently, residual confounding cannot be excluded.

A further limitation relates to the classification framework. Although the annotation framework was informed by previous image-based dietary assessment research, our own past efforts in the domain [26] and refined through expert consensus discussions, it was developed specifically for this study and is not a formally validated dietary quality assessment instrument. Likewise, the binary healthy/unhealthy classification should be regarded as a pragmatic indicator of consistency with dietary recommendations rather than a comprehensive measure of dietary quality. Inter-rater agreement was assessed using a separate but comparable image dataset, and although annotation reliability may not have been identical in the study dataset, the similarity between datasets suggest that the reported agreement provides a reasonable indication of annotation consistency.

The first study period also partly overlapped with the Covid-19 pandemic, which may have influenced individual behaviours despite schools remining open [23]. Finally, the study was conducted in a central area of Stockholm with many nearby food retailers, limiting generalizability to similar urban contexts. More diverse settings should be examined in future research. Despite these limitations, the main findings were consistent across all analyses, with lunches being less healthy outside of school and snacks frequently unhealthy in both settings.

### Implications for future research

This study provides new insights into adolescents’ dietary patterns during school hours in Sweden by using an innovative method that captures both the timing and location of meals, offering an initial understanding of where students eat within the school neighbourhood. The methodological approach also has strong potential for broader application, as it enables systematic mapping of eating patterns across different school settings. To strengthen the generalisability of these findings, future research should therefore include larger and more diverse samples from both urban and rural areas to better understand how different school environments shape dietary behaviours. To further enhance the scalability and analytical output of these methods, integrating automatic content recognition of meal and beverage images should be considered.

Beyond its potential for wider use, the findings of this study highlight several important challenges related to school lunches and snacking during the school day. Addressing these issues will require strategies that encourage students to eat the school lunch, for example, by improving its appeal, while also strengthening its nutritional quality, particularly by reducing the amount of processed meat served. Future research should also focus on developing interventions that improve the food environment surrounding schools, making healthier options more accessible and supporting healthier snacking patterns both inside and outside the school.

## Conclusions

This study shows that despite access to free school meals, students in central Stockholm tend to purchase food and drinks outside of school grounds, where the options are generally less healthy, potentially leading to behaviours like school meal-skipping. Together with the easy access to vendors selling unhealthy food in the school neighbourhood and the frequent consumption of unhealthy snacks both in and out of school, these patterns highlight important public health concerns related to the school food environment. At the same time, this study provides new and detailed insights into adolescents’ dietary patters during the school day, offering a valuable foundation for future research, both for applying the method in larger or more diverse samples and for designing and evaluating interventions that support healthier eating among students.

## Supplementary Information

Supplementary Material 1.

Supplementary Material 2.

Supplementary Material 3.

## Data Availability

The datasets generated and analysed during the current study are not publicly available due to consent restrictions on sharing meal images outside the research group but are available from the corresponding author on reasonable request.
