# Supplementary material for: Exploring dietary patterns during school hours in Swedish adolescent student population, focusing on out-of-school eating and snacking: a repeated cross-sectional study
Source: BMC Public Health. 2026 Sep 22;26:2650. doi: 10.1186/s12889-026-29555-8 (PMC13595745; doi:10.1186/s12889-026-29555-8)
Supplement: Supplementary file 1 [file 12889_2026_29555_MOESM2_ESM.docx]

**Additional file 1: Supplementary Tables**

**Table S1.** 14-item questionnaire about dietary habits

| # | **Question** | **Code** | **Response Format** |
| --- | --- | --- | --- |
| Q1 | How often do you eat meals or snacks that include hamburgers, fries, frozen meals, pizza, kebabs, nuggets/other fried foods, pies? | Fast-food meal | 7-point frequency scale (≥3/day to <1/week or never) |
| Q2 | How often do you eat chips, candy, chocolate, ice cream, cookies, buns, muffins or other pastries? | Sweets and chips snack | 7-point frequency scale (≥3/day to <1/week or never) |
| Q3 | How often do you eat vegetables, legumes (beans, lentils) or root vegetables? Think about the times when you eat a bit more than e.g. a piece of cucumber on the sandwich. | Vegetable intake | 7-point frequency scale (≥3/day to <1/week or never) |
| Q4 | How often do you eat fruit or berries? | Fruit intake | 7-point frequency scale (≥3/day to <1/week or never) |
| Q5 | How often do you drink soda or other sweetened drinks (sugar sweetened/no drinks called zero or light?) | Sugary-drink intake | 5-point frequency scale (≥4/week to never) |
| Q6 | How often do you drink diet soda or other drinks with sweeteners? For example, drinks called zero or light. | Light drink intake | 5-point frequency scale (≥4/week to never) |
| Q7 | How often do you eat fish or seafood as a main course? | Fish intake | 5-point frequency scale (≥4/week to never) |
| Q8 | How often do you eat sausages, store bought meatballs, ham, salami, bacon, kebab meat, hamburger meat or something similar (not vegetarian options)? | Processed meat intake | 7-point frequency scale (≥3/day to <1/week or never) |
| Q9 | How often do you eat whole grain products, such as oatmeal, brown rice/pasta, whole grain bread, and whole grain cereals/muesli? | Whole-grain intake | 7-point frequency scale (≥3/day to <1/week or never) |
| Q10 | How many days per week do you eat the food served at school for lunch? | School lunch frequency | 6-point scale (0 to 5 days) |
| Q11 | How satisfied are you with the food served for lunch at school? | School lunch satisfaction | 5-point satisfaction scale (Not at all to Very satisfied) |
| Q12 | On the days you don't eat the school lunch, where do you eat your lunch? | School lunch replacement | Multiple choice (6 options) |
| Q13 | How many days per week do you buy something to eat outside of school during school hours? For example, during a break? | Meal outside school frequency | 6-point scale (0 to 5 days) |
| Q14 | If so, what do you usually buy during the break? | Outside-school food type | Multiple choice (9 options) |

**Table S2.** Sensitivity analysis accounting for within-participant correlation

| **Analysis** | **OR for unhealthy food,**  **outside vs. inside** | **95% CI** | **p-value** |
| --- | --- | --- | --- |
| All meals | 3.36 | 2.71-4.16 | < 0.001 |
| Lunches | 2.44 | 1.83-3.26 | < 0.001 |
| Snacks | 2.21 | 1.23-3.97 | 0.008 |

**Table S3.** χ^2^ tests of inside-outside meal healthiness across datasets and contribution-exclusion analyses

| **Healthy vs. unhealthy – locations^a^** | **Full dataset**  χ^2^ (p-value) | **Top 10 excluded**  χ^2^ (p-value) | **<2 images excluded**  χ^2^ (p-value) |
| --- | --- | --- | --- |
| **Total Meal** |  |  |  |
| Combined^b^ | 109.6 (< 0.001) | 107.1 (< 0.001) | 101.3 (< 0.001) |
| 1^st^ period | 78.4 (< 0.001) | 79.8 (< 0.001) | 74.2 (< 0.001) |
| 2^nd^ period | 36.5 (< 0.001) | 32.7 (< 0.001) | 32.3 (< 0.001) |
| **Lunch** |  |  |  |
| Combined^b^ | 31.9 (< 0.001) | 39.5 (< 0.001) | 27.5 (< 0.001) |
| 1^st^ period | 22.7 (< 0.001) | 28.5 (< 0.001) | 20.8 (< 0.001) |
| 2^nd^ period | 9.8 (0.002) | 11.6 (< 0.001) | 7.4 (0.007) |
| **Snack** |  |  |  |
| Combined^b^ | 0.4 (0.529) | 0.6 (0.442) | 0.5 (0.478) |
| 1^st^ period | 0.09 (0.767) | 0.04 (0.839) | 0.3 (0.577) |
| 2^nd^ period | 3.3 (0.068) | 2.7 (0.098) | 3.2 (0.075) |

^a^ χ2 tests assessing whether the difference between healthy and unhealthy meals inside vs. outside of school persist when the two datasets are analysed separately.

^b^ Analysis including combined dataset from both the 1st study period (2019-2021) and 2nd study period (2024), part of the main analysis.

**Table S4.** χ^2^ test comparing meal healthiness between 1^st^ and 2^nd^ study-periods with contributor-exclusion analysis

| **Healthy category** | **Full dataset** | | | **Top 10 excluded** | | | **<2 images excluded** | | |
| --- | --- | --- | --- | --- | --- | --- | --- | --- | --- |
|  | 1^st^ (%) | 2^nd^ (%) | p-value | 1^st^ (%) | 2^nd^ (%) | p-value | 1^st^ (%) | 2^nd^ (%) | p-value |
| **All meals** |  |  |  |  |  |  |  |  |  |
| Inside | 60 | 52 | < 0.001 | 61 | 52 | < 0.001 | 59 | 52 | < 0.001 |
| Outside | 38 | 28 | 0.016 | 35 | 27 | 0.078 | 38 | 28 | 0.021 |
| **Lunches** |  |  |  |  |  |  |  |  |  |
| Inside | 60 | 56 | 0.034 | 61 | 56 | 0.009 | 60 | 55 | 0.025 |
| Outside | 42 | 36 | 0.358 | 38 | 33 | 0.482 | 43 | 38 | 0.466 |
| **Snacks** |  |  |  |  |  |  |  |  |  |
| Inside | 37 | 33 | 0.629 | 35 | 33 | 0.863 | 39 | 33 | 0.454 |
| Outside | 35 | 23 | 0.016 | 33 | 23 | 0.074 | 35 | 23 | 0.017 |
| **Meal location^a^**  Inside | 78 | 83 | 0.001 | 80 | 85 | 0.002 | 78 | 83 | 0.001 |

^a^ χ2 tests examining difference between the datasets, regarding meal location (inside vs. outside school).

^b^ The table shows p-values from χ2 tests comparing the proportion of healthy vs. unhealthy meals between the two study periods. For each dataset, the percentage of healthy foods is also reported to illustrate potential differences.

**Table S5**. Distribution of responses for questionnaire items Q11, Q12 and Q14

| **Q11: School lunch satisfaction (n = 156)** | **n** | **%** |
| --- | --- | --- |
| Not satisfied at all | 21 | 13.5 |
| Slightly dissatisfied | 48 | 30.8 |
| Neither satisfied nor dissatisfied | 37 | 23.7 |
| Satisfied | 47 | 30.1 |
| Very satisfied | 3 | 1.9 |
| **Q12: School lunch replacement (n = 162)** | **n** | **%** |
| I always eat school lunch | 66 | 40.7 |
| I bring lunch from home | 22 | 13.6 |
| I buy at a fast-food restaurant | 41 | 25.3 |
| I buy lunch from a grocery store | 35 | 21.6 |
| I buy lunch in a kiosk (e.g., pressbyrån, 7-eleven) | 31 | 19.1 |
| I don’t eat lunch | 49 | 30.2 |
| **Q14: Outside-school food type (n = 157)** | **n** | **%** |
| Fruit or vegetables | 14 | 8.9 |
| Nuts | 7 | 4.5 |
| Sandwich or yoghurt | 24 | 15.3 |
| Candy, ice cream, cookies, or other pastries | 60 | 38.2 |
| Fast-food | 38 | 24.2 |
| Soda or other sweet drink | 26 | 16.6 |
| Energy drink (not coffee) | 46 | 29.3 |
| Coffee or tea | 20 | 12.7 |
| None of the above | 47 | 29.9 |

**Table S6.** χ^2^-test of inside-outside differences across food and drink categories, with contributor-exclusion analysis

| **Category** | **Full Dataset**  χ^2^(p-value) | **Top 10 excluded**  χ^2^(p-value) | **<2 images excluded**  χ^2^(p-value) |
| --- | --- | --- | --- |
| Unhealthy foods | 109.6 (< 0.001) | 107.1 (< 0.001) | 101.3 (< 0.001) |
| Vegetables | 149.1 (< 0.001) | 104.1 (< 0.001) | 143.0 (< 0.001) |
| Red & processed meat | 91.4 (< 0.001) | 64.2 (< 0.001) | 91.4 (< 0.001) |
| Fish & seafood | 39.5 (< 0.001) | 32.2 (< 0.001) | 38.8 (< 0.001) |
| Fruit & berries | 114.4 (< 0.001) | 69.4 (< 0.001) | 110.2 (< 0.001) |
| Whole grains | 10.4 (0.001) | 9.4 (0.002) | 10.1 (0.001) |
| Dairy | 2.9 (0.089) | 1.0 (0.310) | 3.0 (0.085) |
| Not sweetened drink | 346.8 (< 0.001) | 347.9 (< 0.001) | 311.9 (< 0.001) |
| Sugar sweetened drink | 158.5 (< 0.001) | 140.0 (< 0.001) | 143.8 (< 0.001) |
| Artificially sweetened drink | 48.3 (< 0.001) | 55.1 (< 0.001) | 57.9 (< 0.001) |
| Not sure^a^ | (< 0.001) | (< 0.001) | (< 0.001) |

^a^ Fisher’s exact test was used instead of Pearson’s χ2 test, due to categories with expected cell counts < 5.

**Table S7.** Characteristics of the datasets used in the exploratory bias analyses

| **Analysis dataset** | **Participants (n)** | **Images (n)** | **1^st^ period images (%)^a^** | **2^nd^ period images (%)^a^** |
| --- | --- | --- | --- | --- |
| **Meal dataset (full)** | 475 | 3370 | 68.9 | 31.1 |
| Top 10 excluded | 465 | 2944 | 65.4 | 34.6 |
| <2 images excluded | 404 | 3299 | 69.1 | 30.9 |
| **Drink dataset (full)** | 332 | 1487 | 69.8 | 30.2 |
| Top 10 excluded | 322 | 1206 | 66.3 | 33.7 |
| <2 images excluded | 228 | 1383 | 70.1 | 29.9 |

^a^ Percentages represent the distribution of images across study periods within each analysis dataset.
